# Editorial: The nutritional immunological effects and mechanisms of extracts and metabolites based on the homology of medicine and food, volume II

**DOI:** 10.3389/fnut.2025.1575234

**Published:** 2025-02-25

**Authors:** Yongqi Liu, Yi Wu, Guiyan Yang

**Affiliations:** ^1^College of Veterinary Medicine, China Agricultural University, Beijing, China; ^2^MOE Joint International Research Laboratory of Animal Health and Food Safety, College of Veterinary Medicine, Nanjing Agricultural University, Nanjing, China

**Keywords:** medicine and food homology, nutrition, gut microbiota, translational medicine, immunity

With increasing public awareness of healthy lifestyles and evidence-based nutrition, Medicine and Food Homology (MFH), a traditional Chinese concept originating from Huangdi Neijing Taisu, posits that certain edible substances possess both nutritional and medicinal properties. It is written: “Eating on an empty stomach is food, and patients eat it as medicine”, which reflects the idea of “medicine and food homology”. This theory holds that many foods are both food and medicine and that food can prevent diseases just as medicine does.

Many foods are rich in natural active ingredients such as polyphenols, flavonoids, polysaccharides, saponins, alkaloids, and essential oils (1). The health-promoting properties of sweet potato leaves are attributable to their high content of phenolic acids and flavonoids. Notably, chlorogenic acid (CGA) is highlighted as a key bioactive compound in purple sweet potato leaves (2). Supporting this, Zhang Y. et al. demonstrated the role of CGA in megakaryocyte differentiation and platelet production. CGA has been demonstrated to induce the differentiation of human erythroleukemia cells. In a murine model of immune thrombocytopenia (ITP), CGA significantly increased the platelet levels in the peripheral blood. Furthermore, CGA has been shown to regulate the PI3K/AKT signaling pathway during the treatment of ITP. This study suggests CGA emerges as a potential drug candidate for ITP treatment.

Inulin, a mixture of natural fructans, is a type of polysaccharide (3). Zhang K. et al. investigated the effects of two different types of inulin on the development of atherosclerosis. Both types of inulin attenuate the development of atherosclerosis by improving intestinal flora structure and regulating lipid metabolism. Short-chain inulin shows a better therapeutic effect than long-chain inulin. This study suggests the incorporation of dietary inulin as a therapeutic agent for the treatment of atherosclerosis. These extracts and metabolites have been used to enhance immunity, modulate the gut microbiota, treat inflammation, thus protecting the intestine, liver, heart, and brain health, and control cancer-related symptoms.

MFH material is a highly complex biological mixture whose functional value is influenced by many factors. Its true efficacy may be obscured by the co-existence of other substances. By integrating network pharmacology and molecular docking, researchers can analyze drug-disease interactions and identify key MFH components. This facilitates a more comprehensive understanding of the disease mechanism and drug therapy (4). Jin et al. have proposed a novel MFH therapy specifically for the treatment of microvascular angina pectoris (MVA). This therapy is characterized by its multicomponent, multitarget, and multipathway nature, with its components comprising Platycodon grandiflorum, Angelica sinensis, Allium macrostemon, Codonopsis Radix, Astragalus membranaceus, and licorice. This study demonstrates that MFH exerts multiple beneficial effects on MVA, encompassing anti-platelet aggregation, anti-inflammatory, antioxidant, vascular endothelial protection, vascular dilation, and cardiac health.

The gut microbiota has been identified as a crucial player in the gut-brain axis, influencing the physiological state of the host. It directly affects the intestinal integrity and function, and impacts the gut and brain barriers by microbial metabolites. A dysfunctional intestinal barrier can impair brain barrier function by altering the levels of microbial metabolites, potentially causing harmful changes in the physiological and pathological state of the host (5). Additionally, Yoneda et al. reported that the gut microbiome and C18 polyunsaturated fatty acids (PUFAs) from the gut microbiota are associated with the severity of collagen-induced arthritis in mice. This study indicates that C18 PUFA metabolites in plasma or feces could serve as potential biomarkers for the assessment of arthritis severity and dysbiosis.

The MFH shows great potential in translational medicine. However, current studies on the pharmacological effects of MFH materials mostly use animal models and limited clinical validation in heterogeneous human cohorts (6). Therefore, the future directions of MFH research include an in-depth exploration of their bioactive components and pharmacological mechanisms, further clarifying their application value in food. To leverage the distinctive strengths of MFH materials, it is imperative to undertake comprehensive and exhaustive research, to establish a comprehensive and flawless database. A systematic collection, classification, and organization of MFH resources scientifically and rationally is imperative, accompanied by rigorous data verification to ensure the accuracy and comprehensiveness of the resulting database. This foundation will serve as a crucial element for subsequent in-depth research and promote the development of the MFH materials industry.
